# LASSBio-classFLOW: A Semiautomated KNIME Workflow for Classification Model Benchmarking and Virtual Screening

**DOI:** 10.1021/acsomega.6c09298

**Published:** 2026-10-05

**Authors:** Pedro de Sena Murteira Pinheiro, Jefferson Muniz Alves da Silva, Bárbara da Silva Mascarenhas de Jesus, Daniel Alencar Rodrigues, Lídia Moreira Lima

**Affiliations:** † Laboratório de Avaliação e Síntese de Substâncias Bioativas (LASSBio), Programa de Pós-Graduação em Farmacologia e Química Medicinal, Instituto de Ciências Biomédicas, 28125 Universidade Federal do Rio de Janeiro, Av. Carlos Chagas Filho, 373—Cidade Universitária−CCS, 21941-853 Rio de Janeiro, RJ, Brazil; ‡ School of Pharmacy and Biomolecular Sciences, 655732 Royal College of Surgeons in Ireland, 123 St Stephen’s Green, Dublin 2 D02YN77, Ireland

## Abstract

Machine learning-based quantitative structure–activity relationship (ML-QSAR) modeling requires consistent data preparation, validation, and model comparison. We present LASSBio-classFLOW, an open, modular, and semiautomated KNIME workflow for classification-based ligand screening. It integrates basic molecular structure preparation, configurable class assignment, descriptors and fingerprints calculation, dataset partitioning, hyperparameter optimization of nine algorithms, interactive performance evaluation, user-guided model selection, and external-library prediction. As an example, the workflow was evaluated using 1408 ROCK2 compounds from ChEMBL (218 active and 1190 inactive). Models were trained and optimized by fivefold cross-validation and evaluated on a held-out 20% test set. Support vector machine (SVM) and k-nearest neighbors (kNN) provided the most consistent results across both stages. On the held-out set, SVM favored active-class precision (0.861), whereas kNN achieved higher active recall (0.750). LASSBio-classFLOW (available at https://github.com/pedrosenamp/LASSBio-classFLOW_v1.0.git) therefore provides a transparent, reusable environment for benchmarking, comparative ML-QSAR development, and flexible deployment, while preserving user control over model selection and screening decisions.

## Graphical abstract

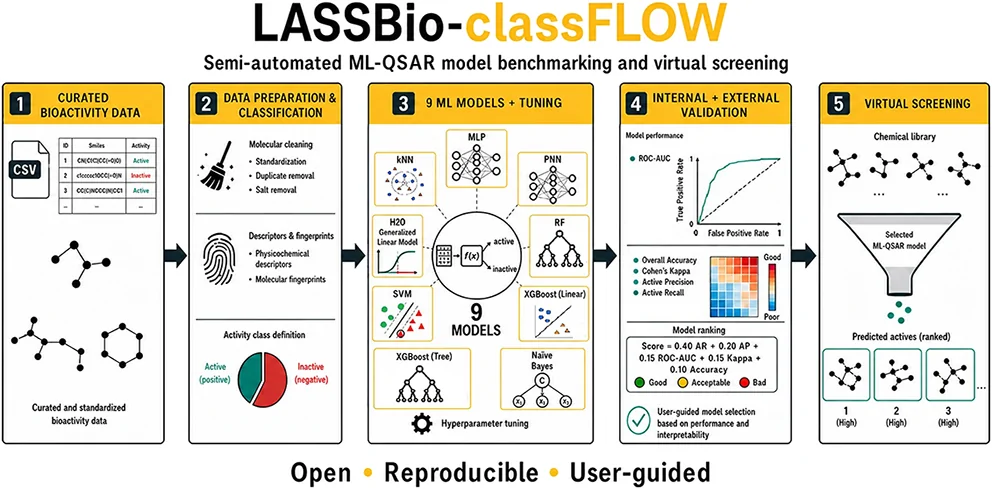

## Introduction

Machine learning (ML) is expanding how chemical data inform molecular discovery, enabling predictive models that support compound prioritization and the exploration of structure–property relationships.^1,2^ Across chemistry and related fields, applications include predicting density functional theory-derived properties of supramolecular structures from lower-cost Hartree–Fock calculations,^3^ recognizing antibiotic residues in milk from electrochemical measurements,^4^ and identifying hydrogel composition from current–voltage responses in programmable soft-matter electronics.^5^ These applications illustrate how ML can relate calculated descriptors or experimental signals to properties and classes of interest.

In drug discovery, the growing availability of curated bioactivity data and open-source cheminformatics tools has made machine learning-based quantitative structure–activity relationship (ML-QSAR) modeling increasingly accessible for compound prioritization. For example, ML classifiers have been used to distinguish active and inactive compounds against cyclin-dependent kinase 2 (CDK2) and prioritize candidates for subsequent molecular docking and molecular dynamics simulations.^2^ Reliability, however, depends on the entire pipeline rather than on the learning algorithm alone, including structure curation, end point and class definition, molecular representation, partitioning, hyperparameter optimization, validation, and model selection. Variation in these choices can hinder comparison and yield different conclusions from the same data, making standardized benchmarks essential.^6,7^

Computational platforms have reduced the technical burden of reproducible QSAR development. KNIME^8^ supports modular visual pipelines in which users can configure reusable operations and inspect intermediate and final results. For example, this architecture underlies TeachOpenCADD-KNIME, which provides code-free access to central CADD tasks; Q-raKtion, which semiautomates curation and integration of PubChem and ChEMBL bioactivity records; and PSW-Designer, which combines ligand design with structure-based virtual screening for photopharmacology.^9–11^ In ML-QSAR, an automated KNIME framework integrated bioactivity data access, molecular representation, feature selection, model construction, and validation.^12^ Other tools address multitask or multitarget classification, small or highly imbalanced data sets, and standardized drug-discovery modeling, whereas QSARtuna, ProQSAR, and RANQSAR extend automation to featurization, benchmarking, uncertainty or applicability-domain assessment, and regression modeling.^13–19^

These platforms demonstrate the demand for accessible and reproducible QSAR tools but differ in scope, interface, supported end points, and degree of automation. This leaves scope for a visual workflow connecting classification-specific data preparation, controlled multialgorithm benchmarking, transparent evaluation, user-guided selection, and external-library prediction. A semiautomated design can standardize repetitive operations while preserving inspection and control, avoiding both opaque full automation and repeated manual reconstruction.

This balance is particularly relevant for imbalanced-classification-based ligand screening. A precision-oriented model may reduce false-positive selection but miss potentially active compounds, whereas a more sensitive classifier may improve active recovery at the cost of a larger experimental set. Model selection should therefore consider complementary class-sensitive metrics and consistency between cross-validation and held-out evaluation, rather than overall accuracy or any single aggregate score.

Herein, we present LASSBio-classFLOW, an open and semiautomated KNIME workflow for constructing, comparing, and applying ML-QSAR classification models in ligand-based virtual screening. It integrates molecular preparation, configurable class assignment and borderline exclusion, RDKit descriptors, user-selected fingerprints, stratified training/test partitioning, hyperparameter optimization of nine algorithms, and an interactive performance comparison. A recall-weighted composite score and sequential rule-based category support interpretation without imposing a universally optimal classifier, while users may forward one or multiple models to an integrated screening module.

We describe the workflow architecture and demonstrate it retrospectively by using ChEMBL bioactivity data for Rho-associated coiled-coil-containing protein kinase 2 (ROCK2). The case study illustrates data preparation, comparative model development, holdout evaluation, and screening-oriented interpretation. The contribution is not a new algorithm or prospective ROCK2 campaign but a reusable visual environment connecting controlled model development, validation, user-guided selection, and external-library classification.

## Results and Discussion

### LASSBio-classFLOW: Workflow Design and Functionality

#### Implementation and General Architecture

LASSBio-classFLOW was developed using the KNIME Analytics Platform. KNIME provides a visual environment in which individual operations are implemented as nodes, and related operations can be encapsulated in reusable components.

The workflow is organized into seven functional sections (Figure 1): (i) data access; (ii) data cleaning and preparation; (iii) data classification; (iv) model training and tuning; (v) export of training and test sets; (vi) visualization and evaluation of models; and (vii) virtual screening of new small molecules. These are described as functional sections rather than strictly sequential stages because data set export and model training form parallel branches after partitioning, whereas virtual screening reuses the preprocessing and molecular-representation operations established during model development.

**Figure 1:**
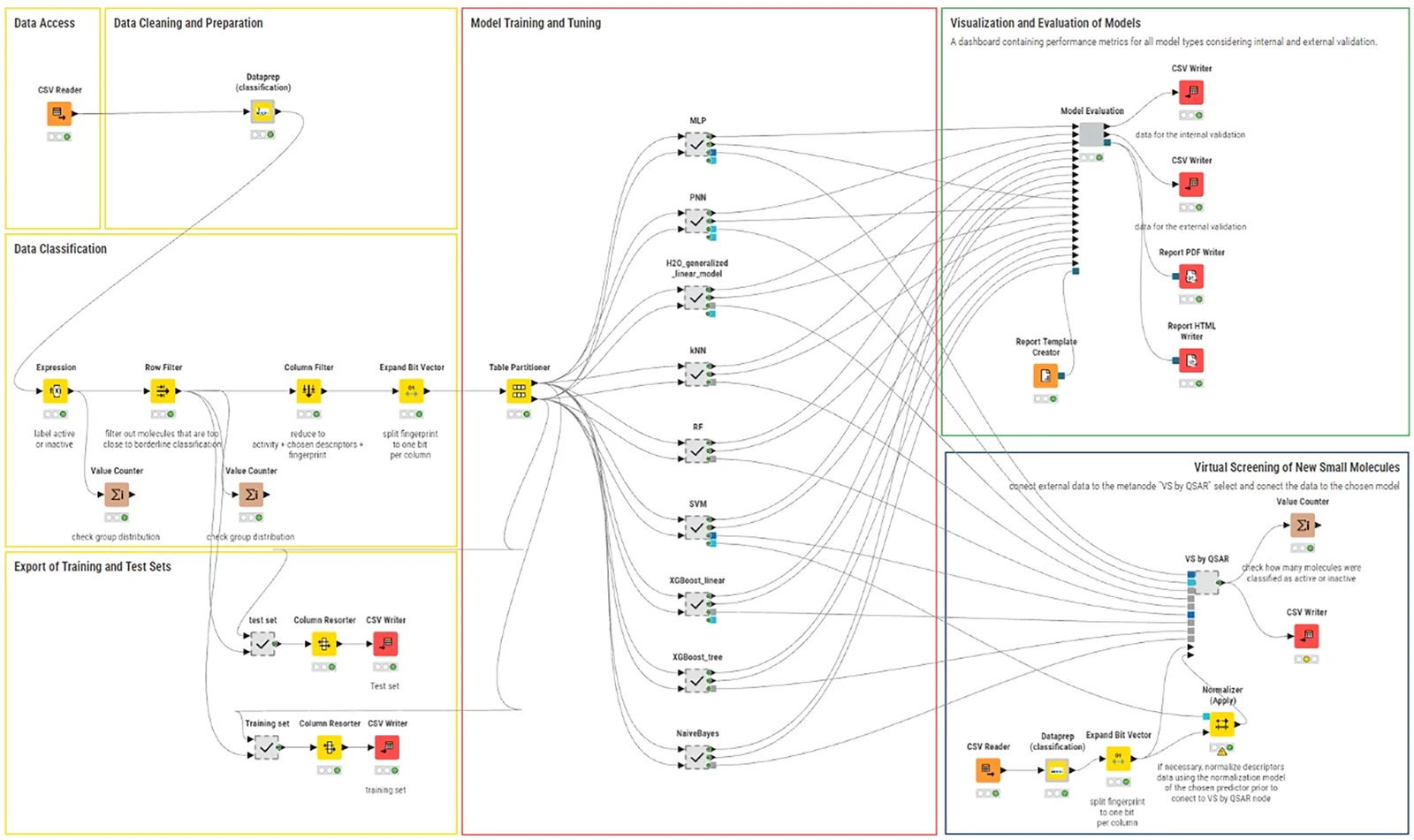
General architecture of LASSBio-classFLOW, comprising seven functional sections: Data Access; Data Cleaning and Preparation; Data Classification; Model Training and Tuning; Export of Training and Test Sets; Visualization and Evaluation of Models; and Virtual Screening of New Small Molecules. Arrows indicate the flow of data, fitted models, and predictions between sections.

User-configurable parameters include the fingerprint type specified in the DataPrep (classification) node, the active/inactive label thresholds defined in the Expression node, an optional borderline-exclusion interval, the training/test ratio, and the model or models applied during virtual screening. This organization semiautomates repetitive operations while retaining user control over decisions that depend on the end point, chemical space, class imbalance, and intended screening objective.

#### Data Access, Cleaning, and Class Assignment

Data access imports a user-provided comma-separated values (CSV) file containing, at minimum, a compound identifier, a SMILES representation, and either an experimental bioactivity value or a previously assigned activity class (active/inactive). The relevant columns are selected by the user, allowing the same workflow to be configured for different biological targets and end point definitions.

During data cleaning and preparation, SMILES strings are parsed into RDKit molecular objects, and structures that cannot be successfully processed are excluded. Disconnected structures were resolved by retaining the largest molecular fragment. The workflow excluded records containing undefined or ambiguous stereochemical features. Duplicate entries are identified based on the processed molecular structures rather than on the database identifiers alone. Users should note that the Dataprep (classification) node performs only basic data preparation and should not be regarded as an automated data curation component. Comprehensive data curation should therefore be performed before the workflow is used. This can be accomplished by using other available open-source KNIME workflows.^10,20^

Data classification converts numerical activity measurements into operational active and inactive labels according to user-defined thresholds. Records within an intermediate interval can be excluded to reduce ambiguity near the class boundary; previously assigned categorical labels can also be accepted. The resulting class distribution is displayed before model development so that the imbalance and the consequences of the selected cutoffs can be inspected. Because threshold definitions are end point- and project-dependent, the workflow does not impose universal definitions of activity or inactivity.

#### Molecular Representation, Dataset Partitioning, and Export

DataPrep (classification) generates each molecular representation by combining RDKit physicochemical, constitutional, and topological descriptors with a user-configurable fingerprint suitable for different chemical spaces and modeling objectives (Figure 2). After class assignment and optional borderline exclusion, the workflow branches into modeling and exporting. In the modeling branch, the column filter removes identifiers, SMILES, and molecular-object columns before the fingerprint is expanded into individual binary columns. The Table Partitioner therefore receives only descriptors, fingerprint bits, and the binary activity label as the response.

**Figure 2:**
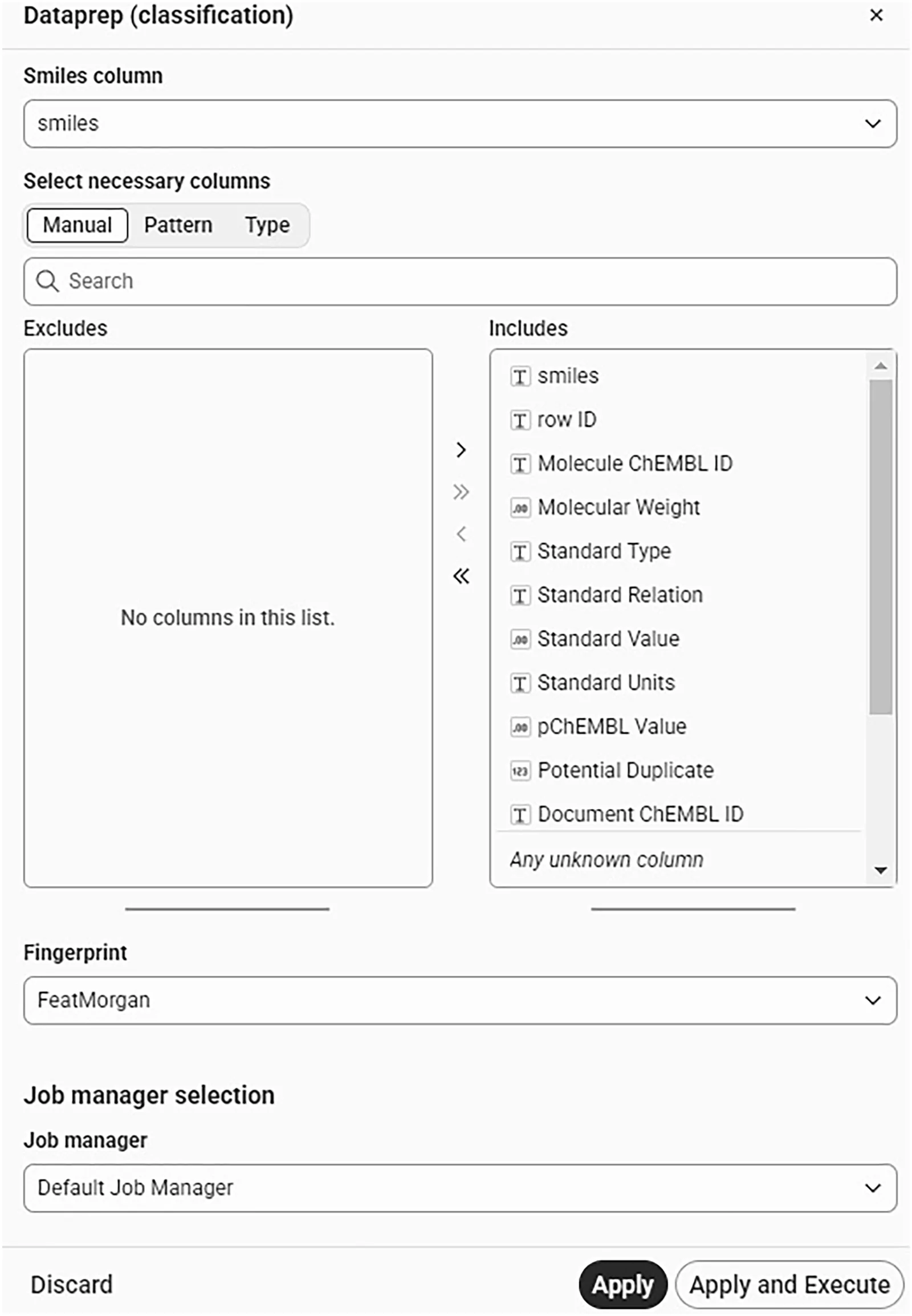
Configuration interface of the Dataprep (classification) node. The node allows users to select the molecular structure column in SMILES format used for descriptor generation, define the input columns to be retained, choose the molecular fingerprint, and specify the job manager responsible for executing the data-preparation workflow. The example shows the use of the FeatMorgan fingerprint.

Descriptor definitions, fingerprint parameters and bit indices, variable names, and column order constitute a feature schema reused unchanged for the training, held-out test, and external-library data, preventing incompatibilities during virtual screening.

Stratified random partitioning uses a user-defined training/test ratio (default 8:2; 80%/20%) and preserves class proportions. In parallel, identifiers, SMILES, structure-related columns, and activity data are mapped to the same partitions and exported in the corresponding training and test CSV files, preserving traceability, auditability, downstream analysis, and reuse of the exact split.

#### Model Training and Hyperparameter Optimization

The same training matrix is supplied in parallel to nine classifiers: multilayer perceptron (MLP), probabilistic neural network (PNN), H2O generalized linear model (H2O-GLM), k-nearest neighbors (kNN), random forest (RF), support vector machine (SVM), XGBoost linear, XGBoost tree, and Naive Bayes.

Tuning is confined to the training set. For each candidate configuration, a Parameter Optimization Loop runs stratified fivefold cross-validation, fitting four folds and predicting the fifth in rotation. Overall accuracy from the aggregated out-of-fold predictions selects the configuration. The selected hyperparameters are propagated outside the tuning loop as flow variables. With these parameters fixed, fivefold cross-validation is rerun solely to generate exportable out-of-fold predictions and internal cross-validation metrics, which cannot be retrieved from within the optimization loop; this second execution performs neither additional tuning nor independent validation. The same parameters are used to fit the final classifier on the complete training set, which is applied once to the held-out test set. Figure 3 illustrates the model training and hyperparameter tuning workflow, using RF as an example. Hyperparameter optimization was performed separately for each learning algorithm. Hyperparameters are predefined settings that control model fitting. The reported intervals indicate the lower and upper bounds evaluated, whereas the step specifies the increment between consecutive candidate values.

- **MLP**. Exhaustive grid search was used to optimize the number of hidden layers (1–5; step size 1) and neurons (5–30; step size 5).
- **PNN**. Exhaustive grid search was used to optimize theta minus (0.1–0.5; step size 0.1) and theta plus (0.2–0.8; step size 0.1).
- **H2O-GLM**. Exhaustive grid search was used to optimize α (0–1; step 0.2) and lambda (0–0.5; step size 0.1).
- **kNN**. Exhaustive grid search was used to optimize the number of nearest neighbors, *k* (3–25; step size 2).
- **RF**. Bayesian optimization based on a tree-structured Parzen estimator (TPE) was used to optimize the number of trees (100–1200) and maximum tree depth (10–30).
- **SVM**. Hill-climbing optimization was applied to an SVM with a polynomial kernel. The optimized hyperparameters were C (1–30; step size 5), kernel power (1–3; step size 1), and γ (0.1–1.0; step size 0.1).
- **XGBoost Linear**. Exhaustive grid search was used to optimize the L2 regularization parameter, lambda (0–5; step size 1), and the L1 regularization parameter, α (0–2; step size 0.5).
- **XGBoost Tree**. Exhaustive grid search was used to optimize max_depth (3–8; step size 1), min_child_weight (1–5; step size 1), subsample (0.6–1.0; step size 0.1), and colsample_bytree (0.6–1.0; step size 0.1).
- **Naive Bayes**. Exhaustive grid search was used to optimize the NBmode parameter (0–2; step size 1).

**Figure 3:**
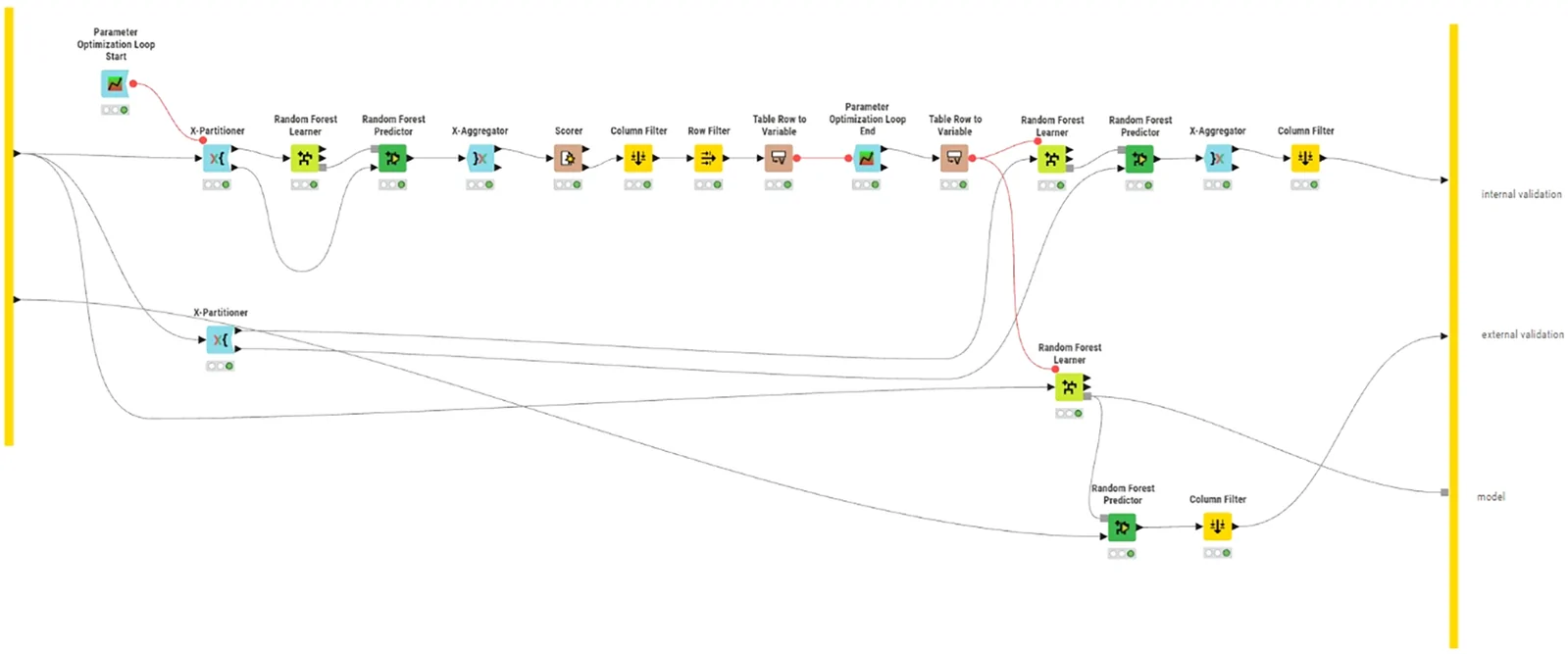
RF model training, hyperparameter tuning, and validation workflow. Hyperparameter optimization is performed using cross-validation within the training set, and the selected parameter values are subsequently used to train the final model. Model performance is assessed through internal validation and evaluation against the held-out external test set, while the optimized trained model is exported for subsequent predictions.

Where used, min–max normalization is fitted to the complete training set before hyperparameter optimization and cross-validation. It is applied only to RDKit descriptors for H2O-GLM, MLP, PNN, SVM, and XGBoost Linear; fingerprint bits remain unchanged. The fitted normalizer is exported and reused for held-out and screening data. Each branch outputs internal cross-validation and held-out predictions and the final classifier, plus the normalizer when applicable. The held-out evaluation estimates generalization within the curated dataset, not to an independently collected chemical series.

#### Interactive Model Evaluation

The Visualization and Evaluation of Models node component summarizes cross-validation and held-out results through overlaid receiver operating characteristic (ROC) curves, heat maps, and sortable tables (Figures 4 and 5). At each stage, the following metrics are displayed and exported in CSV format: ROC-AUC, overall accuracy, Cohen’s kappa coefficient, active precision, and active recall.

**Figure 4:**
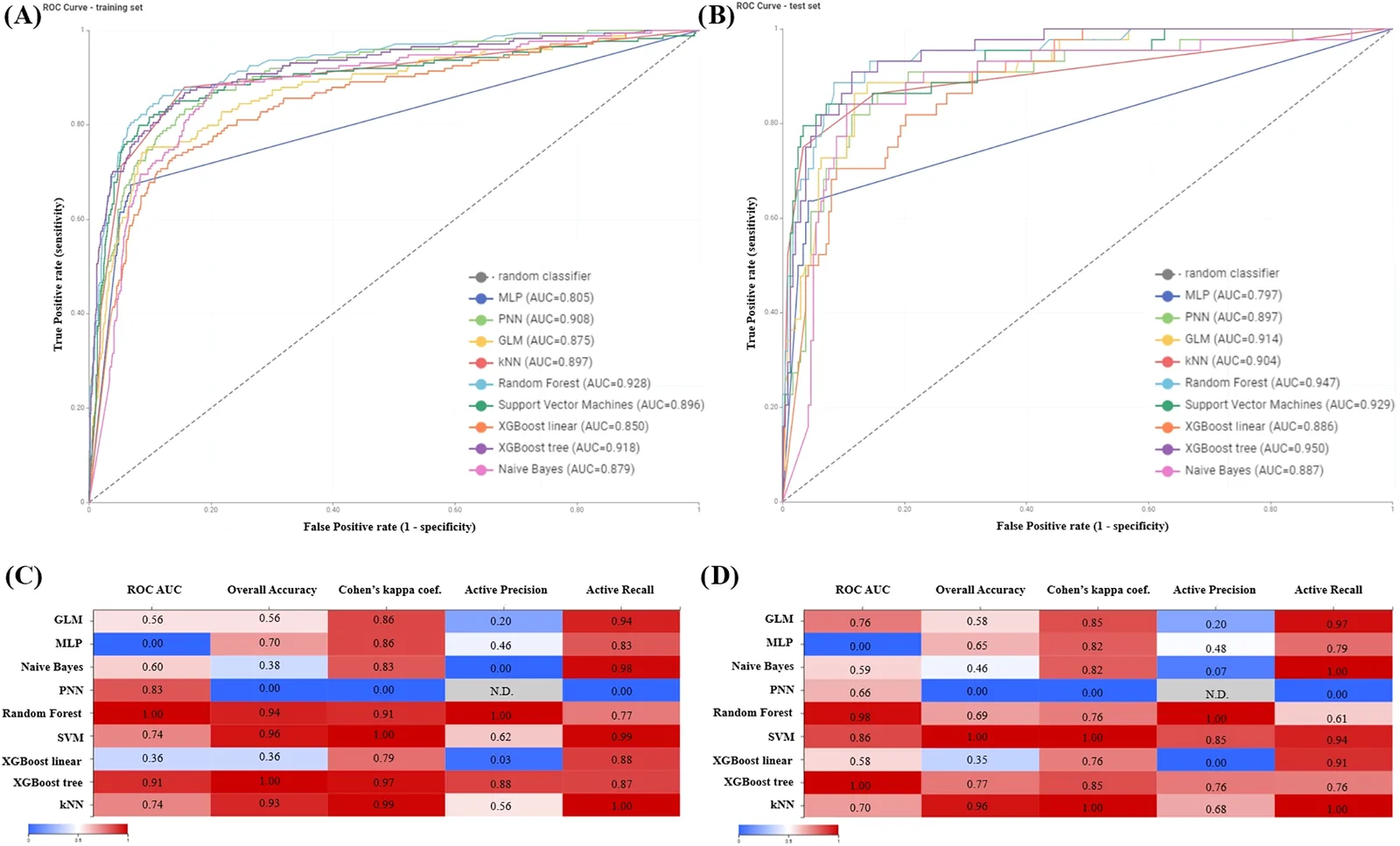
ROCK2 model-comparison outputs. ROC curves for aggregated fivefold cross-validation predictions (A) and the held-out set (B); corresponding heat maps of ROC-AUC, overall accuracy, Cohen’s kappa, active precision, and active recall (C, D, respectively). Cell annotations and colors represent values min–max normalized to the 0–1 range across models, separately for each metric and validation stage. They therefore indicate relative performance within each panel and should not be compared across stages. The original, non-normalized metric values are reported in Figure 5. Gray cells labeled N.D. (not defined) indicate undefined active precision for PNN, which predicted no active compounds.

**Figure 5:**
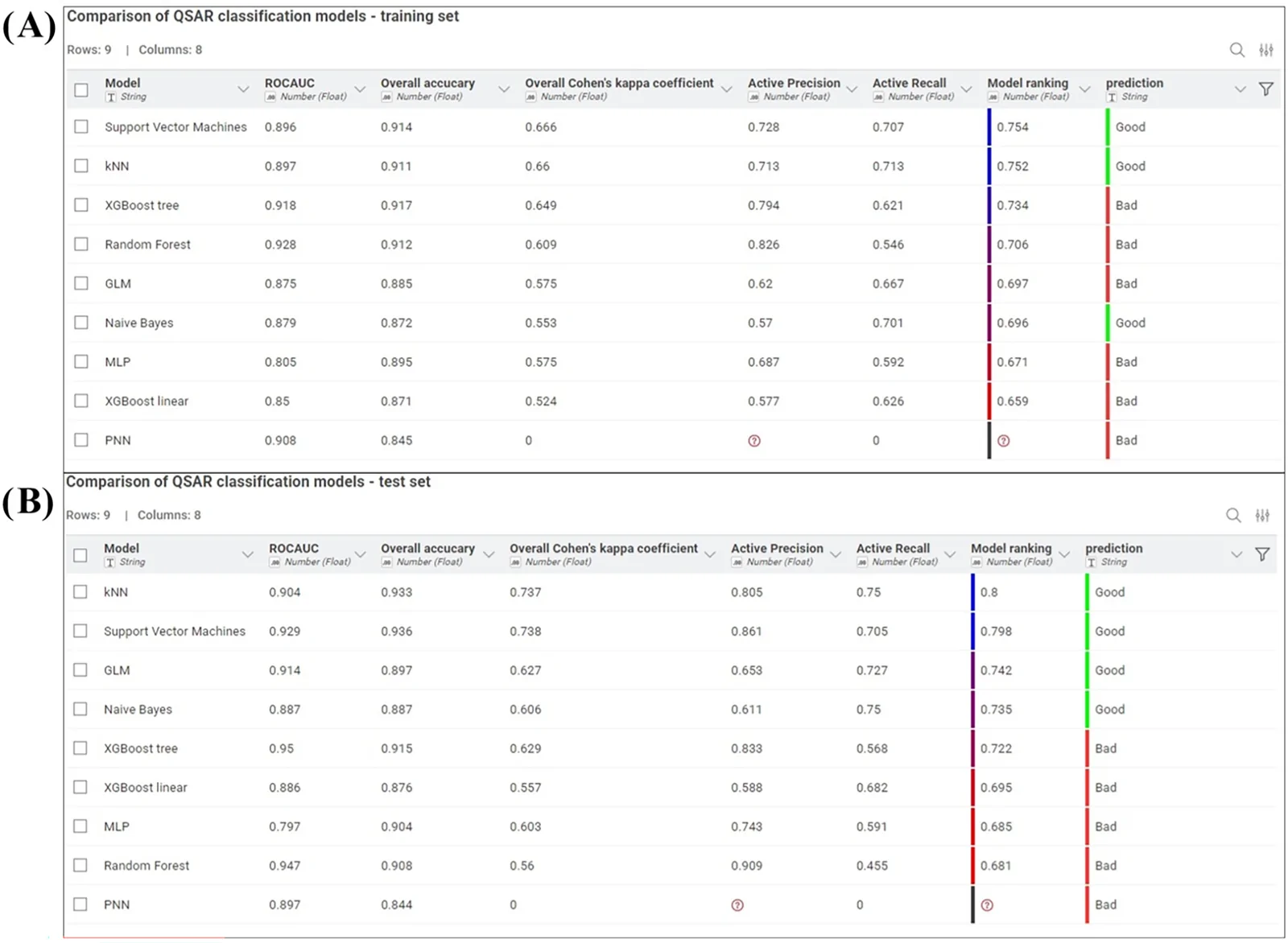
Interactive comparison of the nine ROCK2 classifiers. Metrics from fivefold cross-validation of the 80% training set (A) and application of optimized models to the 20% held-out set (B), ordered by the recall-weighted score. Precision and recall refer to the active class. PNN active precision and score are undefined because it predicted no actives.

The tables also report the recall-weighted score (*Model ranking*)­

modelrankingscore=0.40R_active+0.20P_active+0.15ROC−AUC+0.15κ+0.10Accuracy

where *R*_active and *P*_active denote active-class recall and precision. The score provides a descriptive ranking—not a statistical test or automatic selection criterion—and is not calculated if any component is undefined.

Sequential rules also categorize the model’s quality. “Bad” is assigned if any metric is below its minimum threshold (ROC-AUC < 0.70; accuracy <0.70; Cohen’s κ < 0.40; active precision <0.50; active recall <0.70). Otherwise, “Good” is assigned if any metric exceeds its corresponding threshold (>0.75; >0.75; >0.50; >0.60; >0.75); all remaining models are classified as “Acceptable”. Bad takes precedence. These are operational thresholds, not universal quality standards.

For heat-map visualization, each metric is min–max normalized to the 0–1 range across models within each stage. Colors indicate relative within-panel performance only and cannot be compared across stages; undefined values are shown in gray. Quantitative interpretation uses the untransformed tables.

The workflow does not select a universally optimal classifier. Users may select one or more models for screening based primarily on cross-validation and held-out set performance, ranking score, model category, individual metrics, ROC curves, and project objectives.

#### Virtual Screening of New Small Molecules

External libraries containing compound identifiers and SMILES are processed using the same structural preparation, molecular representation, feature schema, and column order as those used in model development. Model-specific transformations fitted to the training set are then applied.

Users may apply one or more optimized classifiers in a parallel manner. Identifiers, model-specific active/inactive predictions, and their corresponding probabilities are exported for a subsequent analysis.

The current workflow lacks explicit applicability domain analysis; predictions should therefore be interpreted relative to the training chemical space. Applicability domain estimation, probability calibration, and uncertainty quantification are planned developments.

### Application to ROCK2: A Retrospective Case Study

#### Dataset Assembly, Curation, and Class Definition

Human Rho-associated coiled-coil-containing protein kinase 2 (ROCK2) was used as a representative case study. Records retrieved directly from the ChEMBL online database were restricted to standard_type values of “Ki” and “inhibition”, yielding 1712 measurements: 1113 Ki values and 599 percentage-inhibition values.

The end points were processed separately. For Ki data, only records with standard_relation equivalent to “=” were retained, thereby excluding censored measurements. The standard_units field was checked, and Ki values were normalized to nM when necessary. The corresponding pChEMBL values were treated as pKi; after inspection of their distribution, compounds with pKi > 7 (Ki < 100 nM) were labeled active, those with pKi < 7 inactive, and borderline records (6.7 ≤ pKi ≤ 7.0) were excluded. These thresholds are operational rather than universal definitions of ROCK2 activity.

Percentage-inhibition records supplemented only the inactive class: only records reporting less than 30% inhibition were retained and labeled inactive, whereas all remaining inhibition records were discarded. These records were not converted to affinity values nor used to define actives. Because assay concentrations were not considered, these measurements add label uncertainty and do not provide concentration-independent evidence of inactivity.

For duplicate standardized structures, the highest pKi or percentage-inhibition value was retained for the corresponding end point. Curation kept the largest fragment and applied filters for invalid structures and undefined stereochemistry. Sequentially, removal of 231 records with undefined stereochemistry reduced the dataset from 1712 to 1481; duplicate resolution removed 11 additional records, yielding 1470 structures; and exclusion of 62 borderline Ki records produced the final set of 1408 compounds.

The final dataset comprised 1408 unique compounds: 834 derived from Ki records (218 active and 616 inactive) and 574 derived from percentage inhibition records (all inactive). Overall, the dataset contained 218 active and 1190 inactive molecules (15.5% active; inactive-to-active ratio, approximately 5.5:1), requiring class-sensitive metrics in addition to accuracy.

#### Molecular Representation and Modeling Setup

FeatMorgan (radius 2; 2048 bits) was selected to encode feature-based circular environments and recurring pharmacophoric patterns across the heterogeneous ROCK2 set.^21^ Combining 208 RDKit physicochemical, constitutional, and topological descriptors with the expanded fingerprint produced 2256 predictors per compound.

Stratified 80:20 partitioning produced 1126 training compounds (174 active and 952 inactive) and 282 held-out compounds (44 active and 238 inactive). Following the LASSBio-classFLOW protocol, all nine algorithms were optimized on the training set by stratified fivefold cross-validation using overall accuracy.

#### Comparative Performance of the ROCK2 Models

Figures 4 and 5 summarize ROCK2 model performance. Since 84.5% of each subset was inactive, an all-inactive classifier would achieve 0.845 accuracy. PNN showed this failure mode: it predicted no actives, yielding zero active recall and κ despite ROC-AUC values of 0.908 (cross-validation) and 0.897 (held-out); thus, its ranking ability did not translate into useful classifications at the selected threshold.

Cross-validation highlighted distinct profiles: SVM and kNN balanced agreement and active recovery (κ/recall: 0.666/0.707 and 0.660/0.713, respectively), whereas Random Forest and XGBoost Tree traded higher ROC-AUC and precision for lower recall.

The held-out results preserved this pattern: SVM achieved ROC-AUC of 0.929, an accuracy of 0.936, κ of 0.738, precision of 0.861, and recall of 0.705; kNN achieved ROC-AUC of 0.904, an accuracy of 0.933, κ of 0.737, precision of 0.805, and recall of 0.750. XGBoost Tree and Random Forest attained the highest ROC-AUC values (0.950 and 0.947) and high precision (0.833 and 0.909) but recalls of only 0.568 and 0.455. Naive Bayes matched kNN recall (0.750) with lower precision (0.611).

SVM and kNN were effectively tied: their scores were 0.754 and 0.752 in cross-validation and 0.798 and 0.800 on the held-out set, and both were “Good” at both stages. The 0.002 differences do not support meaningful superiority.

Random Forest and XGBoost Tree were “Bad” because recall fell below 0.70; PNN was “Bad”, with an undefined score, because recall and κ were zero and precision was incalculable. These cases show why minimum thresholds complement ranking.

Cross-validation and held-out estimates were broadly consistent, especially for SVM and kNN.

#### Screening-Oriented Model Interpretation and Deployment Options

No classifier was superior for every screening objective. kNN recovered more actives and may be preferable when false negatives are costly, whereas SVM produced more-precise active predictions and may better limit false positives.

The workflow permits either model to be deployed alone or both to be applied in parallel as a consensus approach.

Because compound-level agreement between SVM and kNN was not analyzed, the present study does not evaluate or claim a benefit from combining their predictions.

No prospective library screening was performed; the case study therefore demonstrates retrospective model development and comparison rather than prospective ligand discovery.

## Conclusions and Outlook

LASSBio-classFLOW is an open, modular, and semiautomated KNIME workflow integrating data preparation and class definition, molecular representation, multialgorithm optimization, interactive evaluation, user-guided selection, and external-library screening under a consistent feature schema and validation protocol.

The retrospective ROCK2 case study showed that model choice depends on screening priorities: SVM produced more-precise active predictions, whereas kNN recovered more actives. Neither was universally superior, and the workflow supports the selection of either model according to the intended screening objective.

Interpretation is limited by treating nonharmonized low percentage-inhibition records as inactive, optimizing overall accuracy despite class imbalance, and using a random held-out split from the same curated data set. Applicability domain analysis, probability calibration, and prospective experimental validation were not performed. These limitations provide directions for further development, alongside planned extensions to the workflow.

Future versions of LASSBio-classFLOW will incorporate graph neural network (GNN) models, enabling comparisons between models that learn molecular representations directly from graph structures and the descriptor- and fingerprint-based classifiers evaluated here. Planned modifications also include greater automation within the virtual-screening node to reduce manual intervention when applying trained models to external compound libraries while preserving user control over model selection and screening settings. Another priority is to offer scaffold-based splitting as an alternative to stratified random training/test partitioning, allowing users to assess the predictive performance when compounds sharing the same molecular scaffold are assigned to the same partition. This option would support a more demanding evaluation of the generalization across distinct scaffolds. Further developments will address applicability-domain assessment, probability calibration, and optimization objectives suited to imbalanced data sets, complemented by prospective experimental evaluation of prioritized compounds.

## Experimental Section

### Workflow Implementation and Node Configuration

LASSBio-classFLOW was implemented in KNIME Analytics Platform 5.12 as seven interconnected functional sections: data access; data cleaning and preparation; data classification; model training and tuning; export of training and test sets; visualization and evaluation of models; and virtual screening of new small molecules. Operations used repeatedly or requiring multiple internal nodes were encapsulated as configurable KNIME components. The top-level workflow routes the same partitioned feature matrices to nine model components in parallel and subsequently consolidates their internal validation and held-out predictions in a common evaluation component.

### Data Access

Input data sets were imported using a CSV reader node. Each input table was required to contain, at minimum, a unique compound identifier, a SMILES string, and either a numerical bioactivity end point or a previously assigned activity class. Additional columns could be retained for traceability. File location and column-type assignments were defined in the CSV Reader configuration, whereas the columns used for structure processing and modeling were selected in the downstream Data Prep (classification) component.

### Data Cleaning and Preparation

The CSV reader output was connected to the configurable Dataprep (classification) component. In the component configuration dialog, the SMILES column, the input columns to be retained, the molecular fingerprint type, and the KNIME job manager were specified. The default job manager was used in the present implementation.

Within Dataprep (classification), SMILES strings were converted to RDKit molecular objects. Structures that could not be parsed or processed were removed. For records containing disconnected structures, only the largest molecular fragment was retained, thereby removing the smaller counterions and salt fragments. Records containing undefined stereochemical features were excluded, and duplicate detection was performed by using the processed molecular structures rather than database identifiers.

The RDKit descriptor calculation node calculated all 208 descriptors available under the installed KNIME/RDKit configuration. The component also generated the fingerprints selected by the user. For the ROCK2 application, the fingerprint option was set to FeatMorgan, with a radius of 2, a length of 2048 bits, feature-based atom invariants enabled, and chirality encoding disabled. Data prep (classification) returned the processed structures, retained input columns, calculated descriptors, and fingerprint vector in a single table.

### Data Classification

The output of Dataprep (classification) was passed to an Expression node, which created the Activity response column. Conditional expressions mapped the selected numerical end point to the “active” and “inactive” labels using user-defined thresholds. Values within an optional uncertainty interval were not assigned to either class. For the ROCK2 Ki data, compounds with pKi > 7 were assigned as active, compounds with pKi < 7 were assigned as inactive, and records between 7.0 and 6.7 were excluded. Percentage-inhibition records retained during dataset assembly had already been assigned exclusively to the inactive class.

A Value Counter node connected directly to the Expression output counted the initial occurrences of each activity value, allowing the user to inspect the effect of the class-assignment rules. A row filter subsequently retained only rows assigned as active or inactive and removed records falling within the uncertainty interval or lacking a valid class assignment. A second value counter, configured for the filtered activity column, reported the final class distribution supplied to model development.

A column filter then removed compound identifiers, SMILES strings, RDKit molecular-object columns, and other nonpredictive metadata from the modeling branch. Only the activity response, the selected RDKit descriptors, and the fingerprint vector were retained. The expand bit vector node converted the fingerprint vector into one binary column per bit. For the ROCK2 configuration, the resulting matrix contained 208 RDKit descriptors and 2048 FeatMorgan bits, corresponding to 2256 predictor variables per molecule. No feature selection or dimensionality reduction procedure was applied. The same feature names, bit indices, data types, and column orders were preserved throughout training, held-out evaluation, and external-library prediction.

### Dataset Partitioning and Export of Training and Test Sets

The completed feature matrix was supplied to a table partitioner node configured for relative stratified sampling. The activity column was used as the stratification column, and the dataset was divided once using an 80:20 training/test ratio. The first output contained the 80% training subset, and the second output contained the 20% held-out test subset. For ROCK2, this produced 1126 training compounds, comprising 174 active and 952 inactive molecules, and 282 test compounds, comprising 44 active and 238 inactive molecules.

The two Table Partitioner outputs were simultaneously forwarded to the model components and to the wrapped components labeled training set and test set. In the export branch, the corresponding identifiers, SMILES strings, activity information, and descriptors were restored to each subset. A column resorter established a consistent output-column order, and separate CSV writer nodes exported the training and test tables to user-defined locations. Consequently, every model received the same partition, while the exported files preserved the exact compound membership of each subset.

### Model Training and Tuning

The training and test matrices were connected in parallel to nine dedicated model components: MLP, PNN, H2O-GLM, kNN, RF, SVM, XGBoost linear, XGBoost tree, and Naive Bayes. Each component contained a model-specific learner and predictor together with the nodes required for hyperparameter optimization, cross-validation, scoring, final model fitting, and the held-out prediction. Each component returned the aggregated out-of-fold predictions, held-out test predictions, and the fitted final model. Components requiring descriptor normalization additionally returned the fitted normalization models.

The general optimization sequence consisted of a Parameter Optimization Loop Start, X-Partitioner, model-specific Learner, model-specific Predictor, X-Aggregator, Scorer, Column Filter, Row Filter, Table Row to Variable, and Parameter Optimization Loop End. The X-Partitioner was configured for stratified fivefold cross-validation using activity as the stratification column. For each candidate hyperparameter configuration, four folds were supplied to the learner and the remaining fold was supplied to the predictor. The X-Aggregator concatenated the predictions obtained for five validation folds. The Scorer compared activity with the corresponding prediction column, treating active as the positive class. The overall accuracy calculated from the aggregated out-of-fold predictions was forwarded to the Parameter Optimization Loop End as the objective to be maximized.

The best-performing parameter values were converted into KNIME flow variables by a Table Row to Variable node and propagated to subsequent learner nodes. Because prediction tables generated inside the optimization loop were not retained as final outputs, a second fivefold cross-validation sequence was executed with the selected parameters fixed. This second sequence used an X-Partitioner, the corresponding learner and predictor, and an X-Aggregator to produce one out-of-fold prediction for every training set compound. It performed no additional hyperparameter searches. The resulting aggregated predictions were used to calculate the reported internal validation metrics and confusion matrices.

In parallel, the optimized learner was fitted once to the complete 80% training subset. The resulting model was applied by the corresponding predictor node to the untouched 20% test subset. Column filter nodes retained the experimental activity class, predicted class, and model-specific score or probability assigned to the active class. The fitted classifier was also exposed through a model output port for subsequent use in the virtual screening module.

For algorithms requiring scaled continuous variables, min–max normalization was restricted to the 208 RDKit descriptor columns; the binary fingerprint columns were not transformed. Normalization was used in the H2O-GLM, MLP, PNN, SVM, and XGBoost Linear branches. The following model-specific parameters were evaluated. For MLP, exhaustive grid search evaluated 1–5 hidden layers in increments of 1 and 5–30 neurons in increments of 5. For PNN, theta minus was varied from 0.1 to 0.5 in increments of 0.1, and theta plus from 0.2 to 0.8 in increments of 0.1. For H2O-GLM, α was varied from 0 to 1 in increments of 0.2 and lambda from 0 to 0.5 in increments of 0.1. For kNN, the number of neighbors was varied from 3 to 25 using odd values. RF was optimized by a tree-structured Parzen estimator using 100–1200 trees and maximum tree depths of 10–30. For the polynomial-kernel SVM, hill-climbing optimization evaluated C from 1 to 30 in increments of 5, kernel power from 1 to 3 in increments of 1, and γ from 0.1 to 1.0 in increments of 0.1. XGBoost Linear used exhaustive grid search for lambda from 0 to 5 in increments of 1 and α from 0 to 2 in increments of 0.5. XGBoost Tree evaluated max_depth from 3 to 8, min_child_weight from 1 to 5, subsample from 0.6 to 1.0, and colsample_bytree from 0.6 to 1.0. Naive Bayes evaluated NBmode values from 0 to 2.

### Visualization and Evaluation of Models

The internal-validation and held-out prediction tables generated by the nine model components were connected to the corresponding input ports of the Model Evaluation component. The active class was defined as the positive class. For each model and validation stage, the component calculated ROC-AUC from the continuous active-class score, overall accuracy, Cohen’s kappa coefficient, active-class precision, and active-class recall. Confusion matrices from the aggregated out-of-fold predictions and held-out predictions were retained for Supporting Information. When no compound was predicted as active, active precision and any score depending on it were recorded as undefined.

The component generated overlaid ROC curves, heat maps, and sortable comparison tables separately for internal and held-out evaluation. Heat-map values were obtained by min–max normalization across the nine models independently for each metric and validation stage. This transformation was used only for heat-map visualization, including color assignment and numerical cell annotations; the tables and exported files retained the original metric values.

The comparison table calculated the descriptive model-ranking score as 0.40 (active recall) + 0.20 (active precision) + 0.15 (ROC-AUC) + 0.15 (Cohen’s kappa) + 0.10 (overall accuracy). The score was not calculated when any constituent metric was undefined. Models were categorized sequentially as Bad, Good, or Acceptable. Bad was assigned when any metric was below its minimum threshold: ROC-AUC < 0.70, accuracy <0.70, Cohen’s κ < 0.40, active precision <0.50, or active recall <0.70. Among the remaining models, Good was assigned when at least one metric exceeded its corresponding higher threshold: ROC-AUC > 0.75, accuracy >0.75, Cohen’s κ > 0.50, active precision >0.60, or active recall >0.75. Models satisfying neither rule were categorized as Acceptable, and the Bad rule took precedence.

A report template creator node supplied the reporting template to the model evaluation component. Separate CSV Writer nodes exported the internal and held-out evaluation tables, while Report PDF Writer and Report HTML Writer nodes generated static reports at user-defined output locations. The ranking score and categories were used as comparison aids and did not automatically select classifiers. Deployment decisions were made only after the optimized models had been locked, considering the individual metrics, ROC curves, confusion matrices, and consistency between the internal and held-out performance.

### Virtual Screening of New Small Molecules

The virtual screening branch was implemented as a deployment module and was not executed as a prospective screening campaign in the present study. An external library containing compound identifiers and SMILES strings is imported through a second CSV reader and processed using another instance of Dataprep (classification), configured with the same structure column, retained identifiers, descriptor definitions, fingerprint type, fingerprint parameters, and feature order used during model development. An expand bit vector node converts the generated fingerprint into the original bit-column schema. When required by the selected classifier, a normalizer (apply) node applies the normalization model fitted from the training set.

The resulting feature matrix and the fitted output of one or more model components are connected to the corresponding ports of the VS by the QSAR component. This component applies each connected classifier without retraining and returns the compound identifier, model-specific active/inactive prediction, and associated active class probability. Predictions from multiple classifiers can be generated in parallel but are not automatically combined with a consensus classification. A value counter reports the number of molecules assigned to each predicted class, and a CSV writer exports the complete prediction table for subsequent interpretation.

## Supplementary Material









## Data Availability

The LASSBio-classFLOW workflow, curated ROCK2 dataset, example input files, cross-validation and held-out evaluation outputs, model-optimization settings, dependency information, and user documentation are publicly available at https://github.com/pedrosenamp/LASSBio-classFLOW_v1.0.git.

## Notes

Exact ROCK2 training and test sets used in the case study, including compound identifiers, SMILES strings, activity labels, and calculated RDKit descriptors (CSV)
Complete model-evaluation results for the nine classifiers from internal fivefold cross-validation and held-out test-set evaluation (CSV)
ROC-AUC, overall accuracy, Cohen’s kappa coefficient, active- and inactive-class F-measure, precision, recall/sensitivity, specificity, and confusion-matrix counts (CSV)
True positives, true negatives, false positives, and false negatives for both activity classes (CSV)

## Abbreviations

CADD: computer-aided drug design
CSV: comma-separated values
GLM: generalized linear model
H2O-GLM: H_2_O generalized linear model
HTML: HyperText Markup Language
Ki: inhibition constant
kNN: k-nearest neighbors
KNIME: Konstanz Information Miner
LASSBio: Laboratório de Avaliação e Síntese de Substâncias Bioativas
ML-QSAR: machine learning-based quantitative structure–activity relationship
MLP: multilayer perceptron
PDF: Portable Document Format
pKi: negative base-10 logarithm of the inhibition constant
PNN: probabilistic neural network
QSAR: quantitative structure–activity relationship
RF: random forest
ROC: receiver operating characteristic
ROC-AUC: area under the receiver operating characteristic curve
ROCK2: Rho-associated coiled-coil-containing protein kinase 2
SMILES: simplified molecular-input line-entry system
SVM: support vector machine
TPE: tree-structured Parzen estimator
VS: virtual screening
XGBoost: extreme gradient boosting.
